# Mitogenomic and Phylogenetic Analysis of the Entomopathogenic Fungus *Ophiocordyceps lanpingensis* and Comparative Analysis with Other *Ophiocordyceps* Species

**DOI:** 10.3390/genes14030710

**Published:** 2023-03-14

**Authors:** Shabana Bibi, Dong Wang, Yuanbing Wang, Ghazala Mustafa, Hong Yu

**Affiliations:** 1 Yunnan Herbal Laboratory, College of Ecology and Environmental Sciences, Yunnan University, Kunming 650091, China; 2 The International Joint Research Center for Sustainable Utilization of Cordyceps Bioresources in China and Southeast Asia, Yunnan University, Kunming 650091, China; 3 College of Biological and Agricultural Sciences, Honghe University, Mengzi 661199, China; 4 The Research Center of Cordyceps Development and Utilization of Kunming, Yunnan Herbal Biotech Co. Ltd., Kunming 650106, China; 5 Department of Plant Sciences, Quaid-i-Azam University, Islamabad 45320, Pakistan

**Keywords:** *Ophiocordyceps lanpingensis*, mitochondrial genome, protein-coding genes, tRNA, *Ophiocordyceps sinensis*, medicinal fungus

## Abstract

*Ophiocordyceps lanpingensis (O. lanpingensis)* belongs to the genus *Ophiocordyceps*, which is often found in Yunnan Province, China. This species is pharmacologically important for the treatment of renal disorders induced by oxidative stress and an inadequate immune response. In the present study, the mitogenome of *O. lanpingensis* was determined to be a circular molecule 117,560 bp in length, and to have 31% G + C content and 69% A + T content. This mitogenome comprised 82% of the whole genome that codes for significant genes. The protein-coding regions of the *O. lanpingensis* mitogenome, containing 24 protein-coding genes, were associated with respiratory chain complexes, such as 3 ATP-synthase complex F0 subunits (atp6, atp8, and atp9), 2 complex IV subunits/cytochrome c oxidases (cox2 and cox3), 1 complex III subunit (cob), 4 electron transport complex I subunits/NADH dehydrogenase complex subunits (nad1, nad4, nad5, and nad6), 2 ribosomal RNAs (rns, rnl), and 11 hypothetical/predicted proteins, i.e., orf609, orf495, orf815, orf47, orf150, orf147, orf292, orf127, orf349, orf452, and orf100. It was noted that all genes were positioned on the same strand. Further, 13 mitochondrial genes with respiratory chain complexes, which presented maximum similarity with other fungal species of *Ophiocordyceps*, were investigated. *O. lanpingensis* was compared with previously sequenced species within *Ophiocordycepitaceae*. Comparative analysis indicated that *O. lanpingensis* was more closely related to *O. sinensis*, which is one of the most remarkable and expensive herbs due to its limited availability and the fact that it is difficult to culture. Therefore, *O. lanpingensis* is an important medicinal resource that can be effectively used for medicinal purposes. More extensive metabolomics research is recommended for *O. lanpingensis*.

## 1. Introduction

Mitochondria establish a fully developed eukaryotic cellular system and perform the most important functions in cellular pathways, such as respiration and energy production [1]. A feature that differentiates mitochondria (mt) from the nucleus is the presence of numerous mitochondrial genomes (mitogenomes) that encrypt messages for the coding of a variety of genes to perform different functions [2]. Due to its high copy number and rapidly conserved inherited genetic functions, as well as its fast evolution, mitochondrial DNA (mt-DNA) has been extensively acknowledged as a molecular marker present in the evolutionary trees of a large number of fungi and mushrooms [3]. Through the study of ascomycetes, researchers have discovered that mt-DNAs are usually circular and significantly encoded by the same DNA strand [4]. 

Fungi are a large group of eukaryotic entities that are classified as microorganisms (for example, yeasts, molds, and mushrooms) and are ubiquitously scattered across the globe [5]. In this study, the modern taxonomy of *Cordyceps senu lato* was tabulated, including three families of *Cordyceps s. l*. and their genera, which consist of a number of species. All the species in this study belong to the *Ophiocordycipitaceae* family, which consists of 477 species [6]. The study of medicinal mushrooms and fungal species is highly established in China. Some fungal species are considered traditional Chinese herbs. As the knowledge of Cordyceps has increased and its important secondary metabolites have been screened [7], it has begun to be investigated for its medicinal potential in relation to diabetes, thrombosis, cancer, pathogenic infections, blood pressure management, asthma, and lung and kidney diseases, as well as for its ability to enhance immunity and metabolic activities [8]. 

*O. sinensis*, previously known as *C. sinensis*, is a well-known Chinese caterpillar fungus [9]. Considered an important medicinal fungus, it is widely accepted as being a traditional Chinese herbal medicine and is considered a significant remedy for renal diseases, as well as bronchitis, pneumonia, cough, and asthma [10]. The laboratory investigation of *O. sinensis* has proved its medicinal status, as it may be beneficial for the treatment of several kidney diseases, although its actual mechanism of action is not clearly understood [11]. *O. sinensis* may work as a modulator of the immune response, and may manage oxidative stress, as well as other renal functions [12].

Due to its high significance and extensive medicinal use, the wild resources of *O. sinensis* are frequently diminishing. It requires large-scale production and is artificially cultured, making it difficult to fulfill the medicinal demand for it [13]. *O. lanpingensis* has been identified as an important species of the genus *Ophiocordyceps*; as it is closely related to *O. sinensis*, they might share similar properties [14]. *O. lanpingensis* is an outdated therapeutic option for the handling of renal and urinary abnormalities. However, it has been noted that the chemical compositions of *O. lanpingensis* and *O. sinensis* are both unique, but still seem quite similar to one another [12]. Moreover, *O. lanpingensis*’ easy artificial culture procedure means it may be an alternative to *O. sinensis* that could potentially fulfill current medicinal needs [14]. *O. lanpingensis* can help heal pathological kidney injuries and diminish unusual intensities of the kidney index, creatine kinase, serum creatinine, blood urea nitrogen, and malondialdehyde, as well as increase the cell-mediated immune response and the intensities of immunoglobulin G, glutathione, and superoxide dismutase. *O. lanpingensis* at a high dose has presented more significant results in model animals with acute renal failure than those treated with a low dose of *O. lanpingensis*. *O. lanpingensis* may improve the abnormalities of glycerol-prompted acute kidney injury/acute renal failure in model animals by suppressing oxidative stress and improving the cell-mediated immune response [12].

First, this study was systematically designed. Then, the identification and isolation of *O. lanpingensis* from Yunnan Province, China, were performed, followed by the genome sequencing, assembly, and annotation of its mitogenome. Its gene contents were calculated, its tRNA structures were identified, and its genome organization was studied. Moreover, base pair compositions, calculations of the mitogenome size, the identification of protein-coding genes (PCGs), gene arrangements, and information on amino acids associated with the tRNA gene of *O. lanpingensis* and other *Ophiocordyceps* species were also analyzed. Phylogenetic relationships between *Ophiocordyceps* species were also established based on mt PCGs. Our study of the mitogenome of *O. lanpingensis* may provide a pathway for further investigations into its taxonomic classification, targeted gene/protein pathways, and conservation genetics, which in turn may help to enhance the study of other closely related species within *Ophiocordyceps*. 

## 2. Materials and Methods

### 2.1. Sample Collection and DNA Extraction

**Figure 1:**
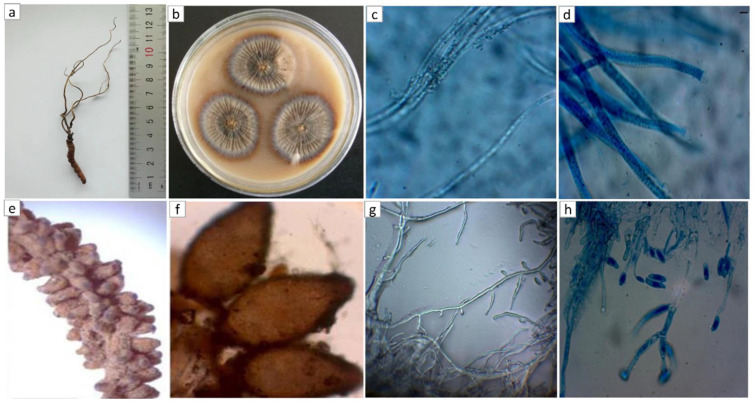
Morphological characteristics of *O. lanpingensis*: the fresh specimen (**a**), colonies (**b**), asci- and ascospores (**c**,**d**), fertile part and perithecia (**e**,**f**), and mycelia and conidia of *O. lanpingensis* (**g**,**h**).

*O. lanpingensis* was isolated from the Biluo Snow Mountains, Yingpan Town, Lanping County, Yunnan Province, China. The *O. lanpingensis* specimen is shown in Figure 1a. Morphological estimations and other molecular assumptions regarding important characteristics of *Ophiocordyceps* have been documented in previous studies [15]. The specimen YHH-OLA0054 of *O. lanpingensis* was deposited in the Yunnan Herbal Herbarium (YHH) of Yunnan University. The strain YFCC 1606 isolated from YHH-OLA0054 was deposited in the Yunnan Fungal Culture Collection (YFCC) at Yunnan University. Mitogenome DNA extraction was performed using a fungal DNA Kit (#D3390-00, Omega Bio-Tek, Norcross, GA, USA), following the manufacturer’s instructions. The integrity of the genomic DNA was calculated with one percent agarose gel electrophoresis application, while the procedural quantitates were calculated using NanoDrop (Wilmington, DE, USA).

### 2.2. Genome Library, Sequencing, Assembly, and Annotation

An Illumina TruSeq library was generated using the IlluminaTruseq™ DNA Sample Preparation Kit (BGI, Shenzhen, China) using the manufacturer’s protocols. Then, genome sequencing was performed on the Illumina HiSeq 4000 platform for the 2 × 150 bp sequencing of paired-end reads. Clean data with no unpaired reads were retrieved, as well as high-quality reads having values < Q20. Then, retrieved clean data were used to assemble the following mitogenomes using the SPAdes 3.13.0 genome assembler [16], and gaps among the contig were filled using MITObim V1.9 [17]. The assembled strain was named YFCC 1606. The coverage was 500X.

### 2.3. Annotation of the Mitogenome

MFannot (http://megasun.bch.umontreal.ca/cgi-bin/mfannot/mfannotInterface.pl) (accessed on 5 February 2022) was used to annotate the mitogenome of *O. lanpingensis*. The MITOS tool [18] also assisted in mitogenome information extraction, and this was designed on the basis of the particular study of the corresponding genetic code (GC). Uncertain results were manually adjusted via sequence alignments with homologous intronless genes from closely related species. The originally annotated *O. lanpingensis* PCG, rRNA, or tRNA genes were also analyzed via alignment with the previously identified *Ophiocordyceps* mitogenome. Mitogenome sequences were scanned and ORF frames were studied using InterProScan software [19]. The tRNAscan-SE 2.0.5 program [20] was used to obtain information on the tRNA genes. Graphical mapping of the mitogenome of *O. lanpingensis* was carried out with the help of the OrganellarGenomeDraw (OGDRAW) tool [21]. Analysis of codon usage (CU) was performed using Sequence Manipulation Suite software [22], based on GC-4. A comparison of the arrangement of genes in *O. lanpingensis* and other reported *Ophiocordyceps* species, as well as genomic synteny estimation, was performed with Mauve v2.4.0 [23].

### 2.4. Mitogenomic Comparison of Six Ophiocordyceps Species

DNASTAR laserene 7.1, an important bioinformatics tool, was used to evaluate the nucleotide base composition of six mitogenomes (http://www.dnastar.com/t-dnastar-lasergene.aspx) (accessed on 1 April 2022). Comparison in terms of nucleotide length, as well as the CU encoding information of RNAs of the complete mitogenomes of *O. lanpingensis*, *O. sinensis*, *Hirsutella vermicola (H. vermicola)*, *H. thompsonii*, *H. rhossiliensis*, and *H. minnesotensis*, was computed using MEGA 6.0 software [24]. Differences in the G-nucleotide base composition and C-nucleotide base composition on the strand disproportionateness were determined using GC and AT skew calculations. These important replication phenomena, wherein prokaryotes present a relative excess of nucleotides, were calculated using the following equations:

AT skew = [A − T]/[A + T]

GC skew = [G − C]/[G + C]

The occurrence of CU was noted using Sequence Manipulation Suite sequence estimating software. The overall mean genetic distances between 15 core protein-coding genes (PCGs) of 6 species were estimated with respect to the Kimura-2-parameter (K2P) substitution model in MEGA 6.0 and using GC [24]. Finally, the synteny of six mitogenomes was performed using the multiple genome alignment application Mauve v2.4.0 [23].

### 2.5. Phylogenetic Analysis

To determine the evolutionary relationship of *Ophiocordyceps*, phylogenetic analysis was conducted using the 14-mitochondrial-PCG dataset, derived from 53 taxa within Ascomycota. Sequence alignment of 14 PCGs was performed using MEGA 6.0. Bayesian interference (BI) was employed to generate the phylogenetic tree based on the 14 concatenated PCGs. The BI analysis was performed with MrBayes v3.1.2 for three million generations using the model GTR + G + I [25]. Trees were sampled every hundredth generation. The initial twenty-five percent of trees were discarded as burn-in, and the remaining trees were used to create a consensus tree using the sumt command. 

## 3. Results

### 3.1. Morphological Characteristics of O. lanpingensis

It is important to reveal the morphological features of *O. lanpingensis* that contribute to the ecology of fungal diversity; therefore, the microscopic features of *O. lanpingensis* were observed in detail in our previous study [26]. The graphical representation of *O. lanpingensis* used in that study is repeated here (Figure 1). *O. lanpingensis* contains several dark-pigmented stromata that are simple and usually fibrous. Its productive (fertile) parts are cylindrical in shape and have a tapered, sterile terminal portion. Its perithecia are oval and apparent, and attach to its stroma at a right angle. Its asci are cylindrical and have a hemispherical ascus cap. Its ascospores are cylindrical and multi-septate, and have indistinct septation (Figure 1). 

### 3.2. Mitogenomic Characteristics of O. lanpingensis

**Figure 2:**
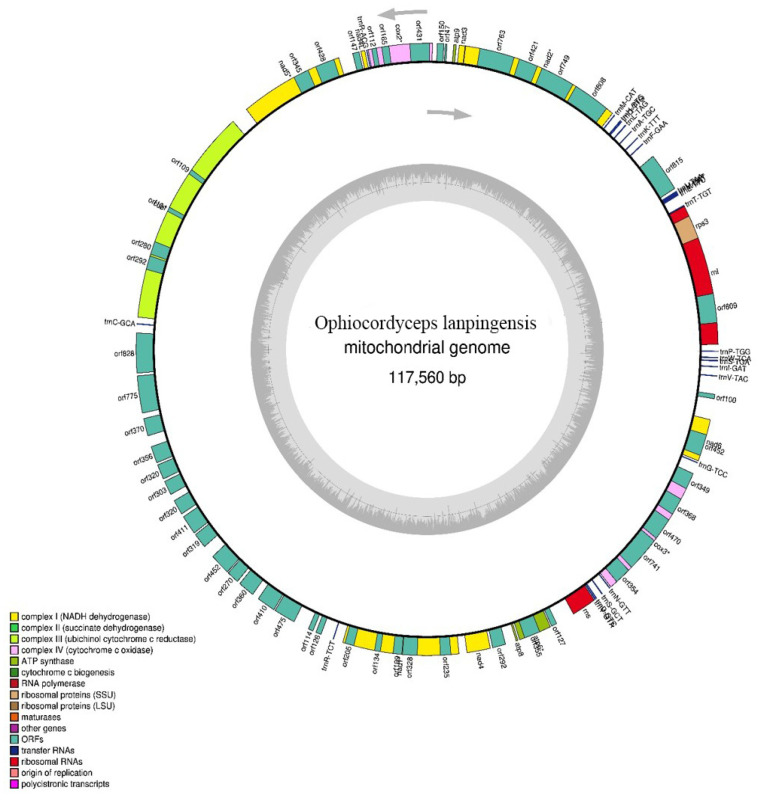
Circular map of the complete mitogenome of *O. lanpingensis* constructed using Organellar Genome DRAW. All functional genes were in the same strand. The mitogenome coordinates had 24 standard protein-coding genes (PCGs), 23 transfer RNA (tRNA) genes, and 2 ribosomal RNA (rRNA) genes.

As it is important to understand the genetic characteristics of *O. lanpingensis*, in this research, the complete mitogenome of *O. lanpingensis* was studied (Table 1). The mitogenome of *O. lanpingensis* was found to be an obvious typical circular molecule 117,560 bp in length (Figure 2). This mitogenome showed similar characteristics to the mitogenome of *O. sinensis*: the complete mitogenome size of *O. sinensis* was found to be 157,510 bp, which was 39,950 bp larger than that of *O. lanpingensis*. Further, the estimated G + C contents of the *O. lanpingensis* mitogenome accounted for as low as 31% of its total contents, which was comparable to *O. sinensis* (30.2%); the A + T contents of *O. lanpingensis* accounted for 69%. The number of PCGs in *O. lanpingensis* was 24 and in *O. sinensis* was 15. Moreover, the G + C contents of the standard PCGs and RNA genes were 23.9% and 35.66%, respectively, in the mitogenome sequence of *O. lanpingensis*. Further, it was estimated that the mitogenome of *O. lanpingensis* was highly complex/compact and that it presented high genome capacity, with 82% of its genome coding for functional gene regions. The number of rRNA/tRNAs (%), which is important for the protein synthesis of *O. lanpingensis*, was found to be 2/23; for *O. sinensis*, this was 2/27.

The functional genes found in the mitogenome of *O. lanpingensis* were associated with respiratory chain (RC) complexes (Table 2), for example, three ATP-synthase complex F0 subunits (atp6, atp8, and atp9); two complex IV subunits/cytochrome c oxidases, i.e., cox2 and cox3; one complex III subunit, i.e., cob; four electron transport complex I subunits/NADH dehydrogenase complex subunits, i.e., nad1, nad4, nad5, and nad6; and eleven ORFs (hypothetical proteins), i.e., orf609, orf495, orf815, orf47, orf150, orf147, orf292, orf127, orf349, orf452, and orf100. These functionally important genes were all found on the same positively oriented strand (Figure 2). It is remarkable that thirteen respective mt genes associated with the RC complexes were conserved and presented the same similarity likelihood as those of other filamentous fungal species [27]. 

The mitogenome of *O. lanpingensis* was found to contain two rRNA genes, rns and rnl, while 25 tRNA genes were detected (as shown in Table 2), coding for a total of 20 amino acids. This enforces the importance of the tRNA ingress phenomenon in relation to the cytoplasm. The presence of tRNA-W was found to be conserved among *O. langpingensis* and *O. sinensis*, which code according to GC-4 (Fox, 1987). Information regarding the genetic components of the mitogenome of *O. langpingensis* is given in Table 3, which presents the genes’ start and stop positions, along with their lengths. 

### 3.3. Codon Usage in the Mitogenome of O. lanpingensis

The CU patterns of the functional mt genes were found to be highly significant with respect to evolutionary and mechanistic analysis [28]. The CU patterns of the important PCGs, such as atp6, atp8, atp9, cob, cox1, cox2, cox3, nad1, nad2, nad3, nad4, nad5, and nad6, were determined. Further, the intronic regions in the intergenic regions in these 14 PCGs, such as atp6 (1 intron), atp8, atp9, cob (5 introns), cox1 (12 introns), cox2 (2 introns), cox3 (4 introns), nad1 (3 introns), nad2 (5 introns), nad3, nad4, nad4L, and nad5 (3 introns), and in the 11 ORFs (orf609, orf495, orf815, orf47, orf150, orf147, orf292, orf127, orf349, orf452, and orf100), were also determined. It was observed that in the case of the 11 ORFs, the start codon AUG most frequently appeared. Further, only orf47 started with “UCU”, only orf150 started with “UAU”, and only orf452 started with “UUU” (Table 4). 

**Figure 3:**
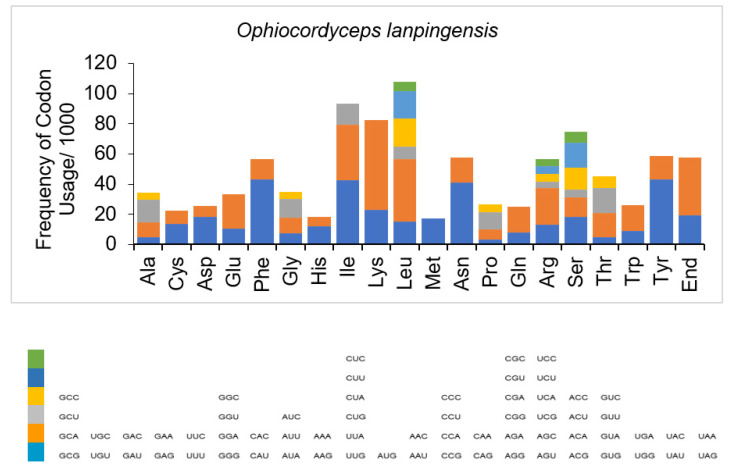
Codon usage analysis of the mitogenome of *O. lanpingensis*. The frequency of codon usage is plotted on the y axis.

It was observed that the most frequently used codon among the PCGs was leucine, followed by isoleucine and lysine (Table 4). The codons for serine, arginine, tyrosine, asparagine, and phenylalanine, as well as the stop codon, were also represented frequently, whereas histidine and methionine were the least used codons (Table 4 and Figure 3). It was determined that the most often used codons were UAA for leucine, AUU for isoleucine, AAA for lysine, AGU for serine, AGA for arginine, UAU for tyrosine, AAU for asparagine, UUU for phenylalanine, and CAU for histidine. UAA and UAG were the only stop codons represented in the mitogenome (Figure 3). 

### 3.4. Transfer RNAs and Ribosomal RNAs

**Figure 4:**
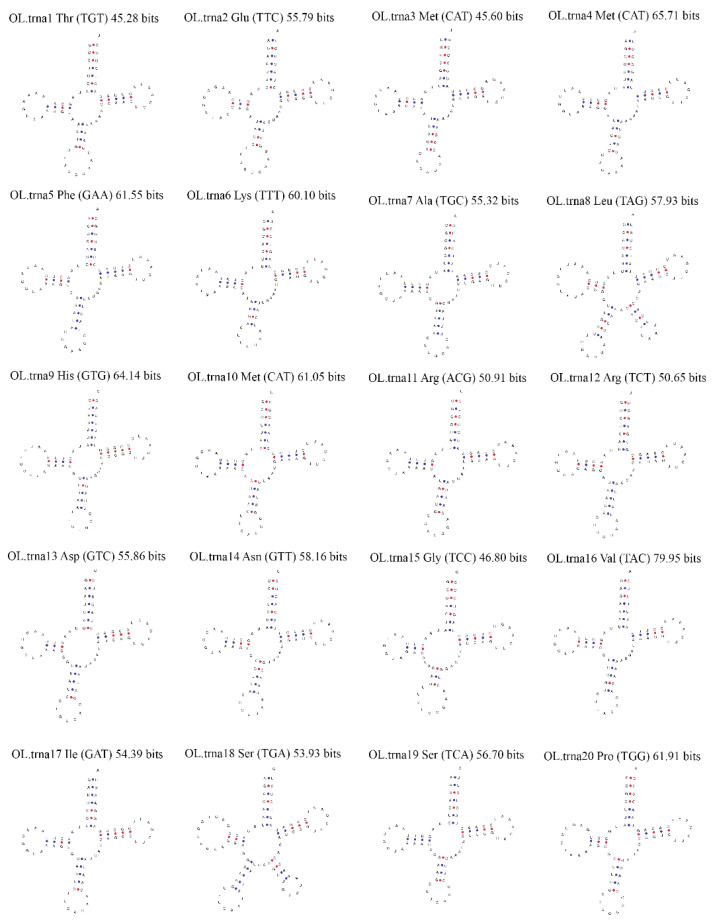
Cloverleaf representation of predicted secondary structures of tRNA genes from the *O. lanpingensis* mitogenome. Each tRNA is labeled with the abbreviation of its corresponding amino acid. The tRNA structural plots were generated using MITOS software (http://mitos.bioinf.uni-leipzig.de/index.py).

tRNA genes are useful as linkers between mRNA and proteins and are involved in the translation process [29]. tRNAscan–SE, a widely accepted tool for predicting tRNA genes in mitogenomes, was used in this study to predict the tRNA genes in the mitogenome of *O. lanpingensis*. A cloverleaf representation of each tRNA was obtained, and 20 tRNAs were found, which, through rough clustering estimation, were divided into two groups of 14 amino acids within the range of 70 to 84 bp in length. The output showed that, among the predicted tRNAs, few were found in the form of multiple copies; the trnM-CAT gene for methionine presented three copies, while the rest of the determined tRNA structures showed only one copy, as shown in Table 5 and Figure 4. 

### 3.5. Mitogenomic Comparison of Six Ophiocordyceps Species

**Figure 5:**
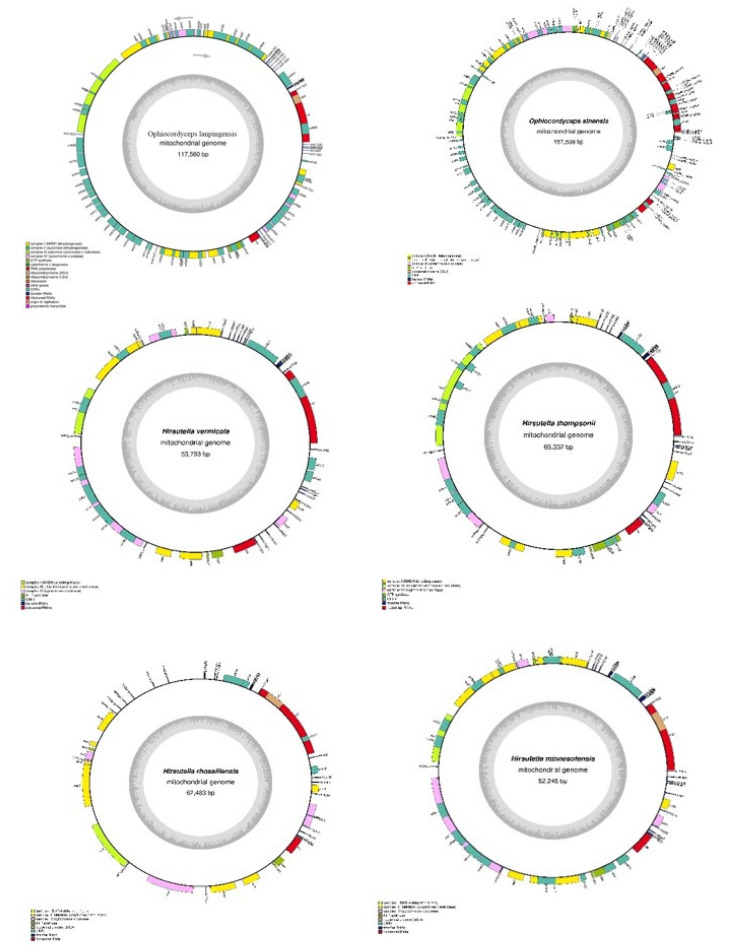
Circle maps of mitogenomes of *O. lanpingensis* and five related species. Genes are represented by differently colored blocks, as indicated in the legend below the maps.

The complete mitogenomes of six *Ophiocordyceps* species were typical circular molecules with total lengths ranging from 52,245 to 157,509 bp (Figure 5). The mitogenome sizes varied widely, with the 157,509 bp mitogenome of *O. sinensis* being the largest, while that of *H. minnesotensis*, at 52,245 bp, was the smallest. The gene-coding regions of these mitogenomes contained genes for rnL, ATP6, ATP8, ATP9, NAD2, NAD3, NAD4, NAD5, and COX2, which are involved in energy metabolism. These were evolutionarily conserved among all the mitogenomes, revealing positive evolutionary selection for these genes. Genes for COX1 were absent only in *H. minessotensis*. Similarly, rns, COX3, and NAD6 were commonly conserved among all the species except *O. sinensis*. NAD1 was found to be unique to *O. lanpingensis*. In addition, several ORFs were identified in the six mitogenomes, with their number ranging from 4 to 66 across the genomes. The smallest number of ORFs was found in *H. minnesotensis*, whereas the highest number was found in *O. sinensis*. All the genes were located on the positive or forward strand, except for ORF 12, ORF 352, and ORF 481 in *O. sinensis*, which were located on the reverse strand.

### 3.6. Codon Usage Analysis of the Mitogenomes from Six Ophiocordyceps Species

**Figure 6:**
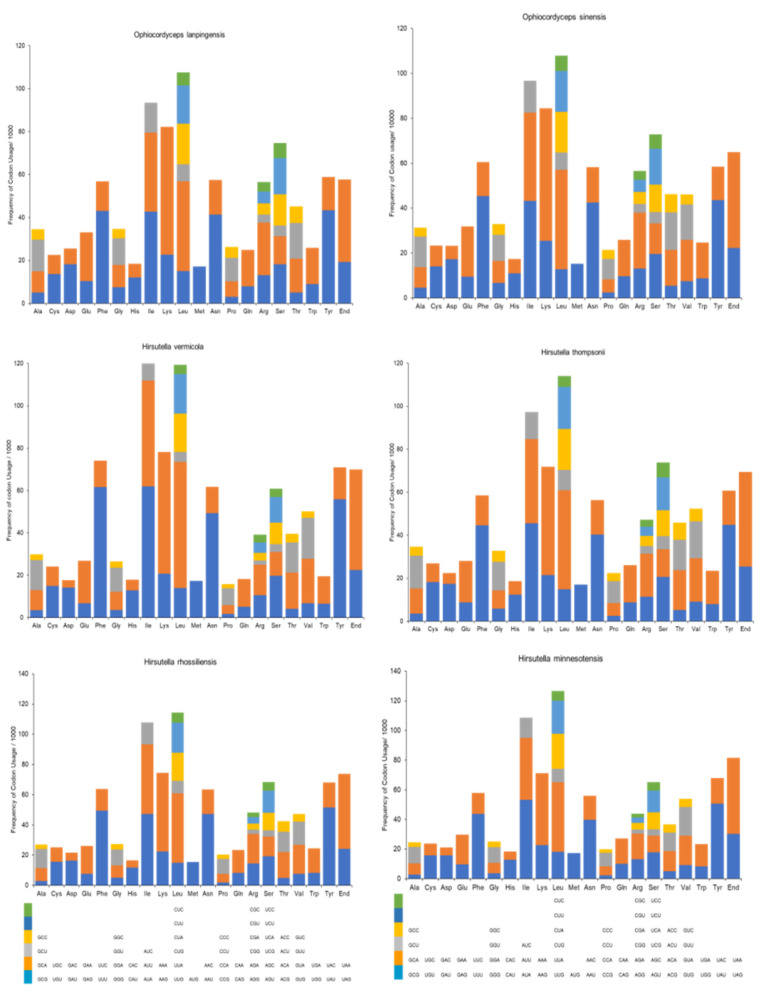
Codon usage analysis of the mitogenomes from six *Ophiocordyceps* species. Frequency of codon usage is plotted on the y axis.

Next, the CU analyses of the six mitogenomes, which are shown in Figure 6, were compared. Leucine was the most frequently coded region, followed by isoleucine and lysine. However, *O. lanpingensis* and *O. sinensis* clearly exhibited similar patterns of CU frequency compared to the other four mitogenomes. 

### 3.7. Gene Arrangement Analysis of Six Ophiocordyceps Species

**Figure 7:**
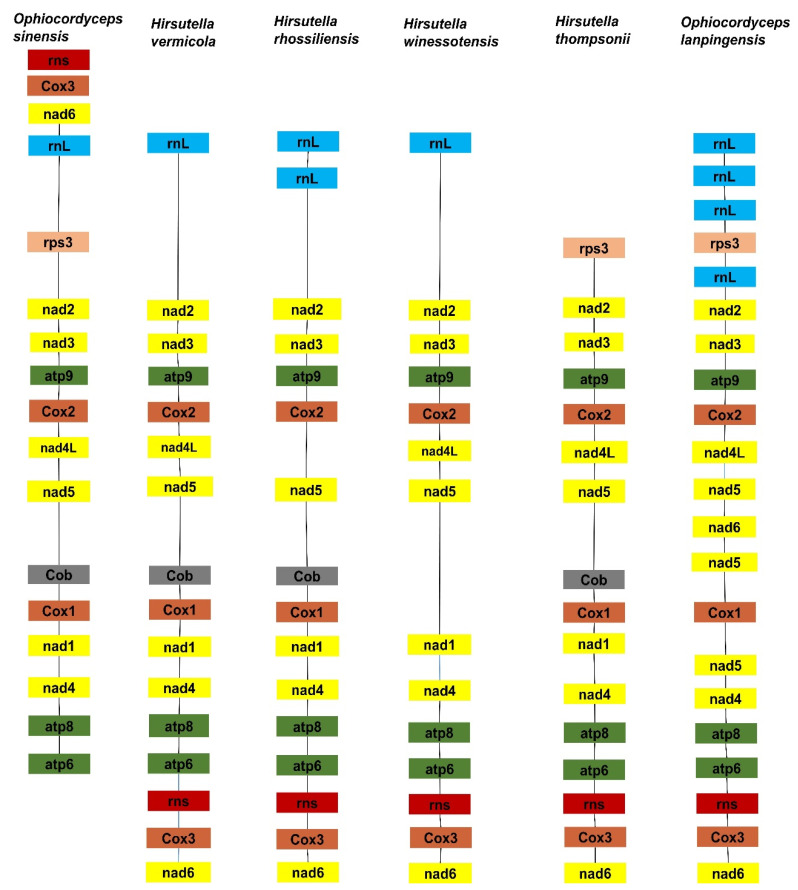
The gene arrangement of mitogenomes of *O. lanpingensis* and other related species.

Gene order and rearrangement in mitogenomes have great significance for biological evolution. In this study, each of the four *Ophiocordyceps* mitogenomes contained two rRNA genes, the small subunit ribosomal RNA gene (rns) and the large subunit ribosomal RNA gene (rnl). There were no significant differences in the number of rRNA genes among the six species, suggesting that these genes are experiencing positive evolutionary selection pressures. Five different groups of gene arrangement were detected in the six *Ophiocordyceps* mitogenomes (Figure 7). The results indicate that the *Ophiocordyceps* species had undergone large-scale gene rearrangements in their mitogenomes during evolution. Large-scale gene rearrangements were observed in the mitogenome of *H. thompsonni*, another *Ophiocordycipitaceae* species. The mt gene order of *O. lanpingensis* was very much like that of *O. sinensis*. Hence, the gene order and sequence homology of these two species, *O. lanpingensis* and *O. sinensis*, revealed that they are experiencing positive natural selection pressures and are closely related. Their sequences and structural homology suggest that they are similar in function. 

### 3.8. Synteny Analysis among Six Ophiocordyceps Species

**Figure 8:**
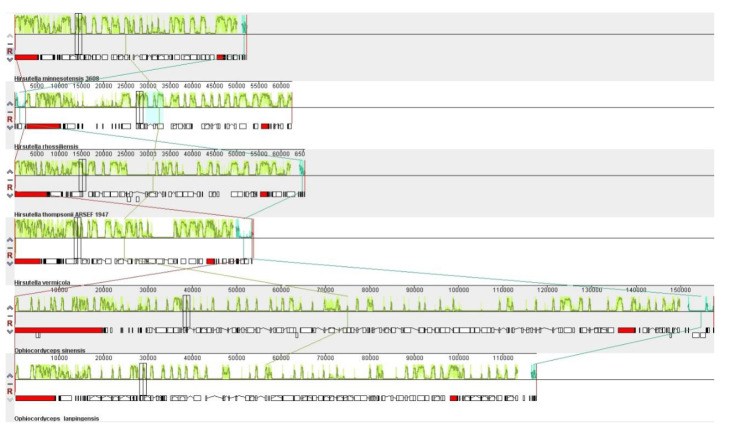
Mitogenome collinearity /synteny progress of six *Ophiocordyceps* species. Mauve alignment determined the homologous regions of the six mitogenomes, which are connected by colored lines to explain the sequence of gene arrangement. The sizes and relative positions of the homologous fragments varied across the mitogenomes.

Synteny analysis indicated that the six *Ophiocordyceps* mitogenomes were divisible into different homologous regions, where the sizes and relative positions of these regions were highly variable (Figure 8). The locations and sizes of these homologous regions varied among the six mitogenomes. In Figure 8, the homologous regions are connected via colored lines, which in turn connect the six mitogenomes, whereas black bar boxes show the genes with similar orders and sequence conservation among the six mitogenomes. Gene order and synteny analysis revealed that 13 genes were conserved across 6 mitogenomes. Meanwhile, the gene orders of *O. lanpingensis* and *O. sinensis* were highly conserved compared to the other four species. 

### 3.9. Phylogenetic Analysis of Six Ophiocordyceps Species

The *O. lanpingensis* mitogenome data were compared with previously published data on other fungal mitogenomes to comprehensively investigate the evolutionary relationships of the fungal species studied. The mitogenome of *O. lanpingensis*, along with five other mitogenomes, namely those of *O. sinensis*, *H. minnesotensis*, *H. vermicola*, *H. rhossiliensis*, and *H. thomsonii*, is presented in the *Ophiocordycipitaceae* family of the BI phylogenetic tree (section highlighted in gray). This tree was constructed to scale, with branch lengths in the same units as those of the evolutionary distances being used to infer the tree. The evolutionary distances were calculated using the maximum composite likelihood technique and are expressed in terms of the number of base substitutions per site. This analysis involved six nucleotide sequences. The positions of codons were estimated using the 1st + 2nd + 3rd + Noncoding formula. It was confirmed that all uncertain positions were discarded against each sequence pair. There was a total of 157,539 positions in the final dataset. Phylogenetic evolutionary investigations were performed using Clustal X2.0 and MEGA 6.0 software [24]. 

**Figure 9:**
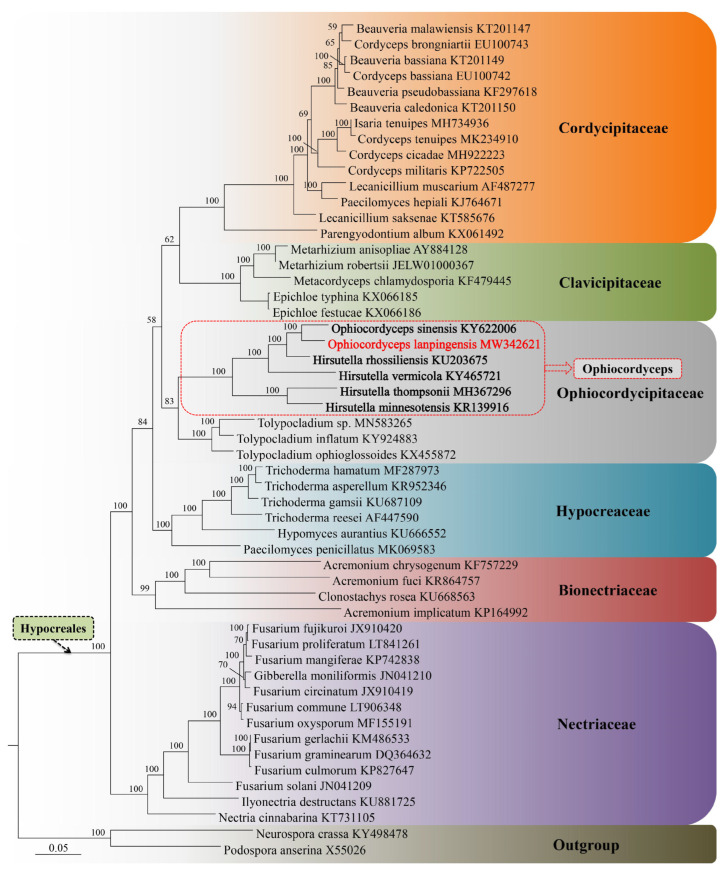
Phylogenetic placement of *O. lanpingensis* in the order Hypocreales, inferred from mitogenomes based on Bayesian inference.

In this study, the sequence alignment and phylogenetic analysis were followed by the analysis of the parameters explained by Wang et al., 2020 [15]. The phylogenetic tree constructed using BI showed that *O. lanpingensis* shares its lineage with *O. sinensis*. As shown in Figure 9, regarding the evolutionary aspects of the fungal mitogenomes, the analysis indicated that *O. lanpingensis* and *O. sinensis* were more closely related than other species. *O. lanpingensis* and *O. sinensis* have previously been placed within the family *Ophiocordycipitaceae*, which includes species such as *H. rhossiliensis*, *H. vermicola*, *H. thompsonii*, and *H. minnesotensis* (Figure 9). Meanwhile, species in the *Clavicipitaceae* and *Cordycipitaceae* families have evolved at a higher evolutionary rate with regard to their mitochondrial genes than *Ophiocordycipitaceae*. 

## 4. Discussion

Mitogenomic analysis has been reported to be a successful technique in several studies, and has demonstrated its benefits for the phylogenetic classification and estimation of evolutionary relationships in rapidly growing populations [30]. Through the increase in genomic knowledge and the advanced technology of next-generation sequencing, the sequencing procedures for the mitogenomes of fungal species have been enhanced, although the investigation of fungal mitogenomes remains more difficult than that of plants and animals [31]. As of July 2019, only 612 mitogenomes of fungi had been reported in the NCBI repository, and 488 of the 612 mitogenomes were from Ascomycetes species [32]. Therefore, it is highly recommended more mitogenomes of fungal species are explored in order to gain a deeper understanding of the fungal kingdom and its evolutionary significance.

In the present study, the first mitogenome of the important species *O. lanpingensis* was sequenced and analyzed in order to reveal its particular characteristics. During the comparative analysis of six *Ophiocordyceps* mitogenomes, it was observed that the size of the *O. sinensis* mitogenome was the largest, at 157,539 bp [33], while the *O. lanpingensis* mitogenome at 117,560 bp was the next most complex and largest *Ophiocordyceps* mitogenome. Further, the nucleotide lengths and CU of the complete mitogenomes of *O. lanpingensis*, *O. sinensis*, *H. vermicola*, *H. thompsonii*, *H. rhossiliensis*, and *H. minnesotensis* were computed, and the results show that leucine was the most frequently coded region among the mitogenomes, followed by isoleucine and lysine. However, *O. lanpingensis* and *O. sinensis* clearly exhibited similar patterns of CU frequency compared to the other four mitogenomes. A total of 25 tRNA genes were retrieved via the *O. lanpingensis* mitogenome analysis, and these were found to play significant roles in decoding mRNAs into proteins, following the translation mechanism. For the very first time, in this study, the complete mitogenome of *O. lanpingensis* was described. It was found to be a circular molecule 117,560 bp in length; its overall G + C content was 31%, while its A + T content was 69%. In addition, the G + C content of *O. sinensis* was found to be 30.8% and its A + T content was about 71%. These values are therefore highly similar among these two species. The mitogenome of *O. lanpingensis* was found to be a generally compacted and very bulky genome, with 82% of the genome-coding region being for functional genes. It was observed that the protein-coding regions of the whole mitogenome encompassed the genes associated with RC complexes, and these genes were all located on the same strand with a clockwise orientation. Thirteen representative mt genes were involved in the RC complexes, showing maximum similarity with species in the filamentous fungus group. Further, *O. lanpingensis* was compared with previously sequenced species within *Ophiocordycepitaceae*. Comparative analysis was used to compare the gene order, synteny, and circular genome arrangements of these species. This analysis indicated that *O. lanpingensis* shares more similar features with *O. sinensis* than the other species.

Four complex 1 (NADH) genes were found to be expressed in *O. langpingensis,* while three genes, namely nad1, nad6, and nad4L, were expressed in *O. sinensis*. One complex III (ubiquinol cytochrome c reductase) gene (cob) was common in both species; three complex IV (cytochrome oxidase) genes, i.e., cox1, cox2, and cox3, were common in both species; three ATP synthase genes, i.e., atp6, atp8, and atp9, were also common in both species. Two ribosomal RNAs (rRNAs) were also common in both species. The tRNA genes of the fungal mitogenomes studied have been proposed to play an important part in the transcription of novel repeats and in the recombination proceedings [34]. Therefore, in this study, 25 tRNAs in *O. lanpingensis* were found and analyzed, while 27 tRNAs were found to be expressed in *O. sinensis* in a previous study [35]. Based on a combined gene dataset (14 PCGs), the phylogenetic information of *Ophiocordyceps* was constructed using Bayesian inference. *O. lanpingensis* has been assigned as a sister species to *O. sinensis*. In this study, it was observed that *O. lanpingensis* and *O. sinensis* share a lineage with *H. rhossiliensis* and *H. vermicola*, while *H. thompsonii* and *H. minnesotensis* form a separate clade (Figure 9). 

## 5. Conclusions

*O. lanpingensis*, a species of the genus *Ophiocordyceps*, is commonly found in Yunnan Province, China. It is pharmacologically important in the treatment of renal disorders induced by oxidative stress and inadequate immune response. In this study, the complete mitogenome of *O. lanpingensis* was described. It was found to be a circular molecule 117,560 bp in length, with its overall G + C content being 31% and its A + T content being 69%. This mitogenome was found to be a generally compact and high-capacity genome, with 82% of its genome coding for functional genes. The mitogenome was found to consist of 20 amino acids, while 24 PCGs were identified within it. The protein-coding regions of the whole mitogenome were found to contain genes involved in RC complexes, such as three ATP-synthase complex F0 subunits (atp6, atp8, and atp9), two complex IV subunits/cytochrome c oxidases (cox2 and cox3), one complex III subunit (cob), four electron transport complex I subunits/NADH dehydrogenase complex subunits (nad1, nad4, nad5, and nad6), one ribosomal RNA (rns, rnl), and eleven hypothetical proteins (orf609, orf495, orf815, orf47, orf150, orf147, orf292, orf127, orf349, orf452, and orf100). These genes were all located on the same strand. Thirteen representative mt genes were found to be involved in the RC complexes, showing high degrees of similarity with those of other species of filamentous fungi. Further, *O. lanpingensis* was compared with previously sequenced species within *Ophiocordycepitaceae*. Comparative analysis was used to compare the gene order, synteny, and circular genome arrangements of these species. This analysis showed that *O*. *lanpingensis* is more closely related to *O. sinensis* than to the other species. *O. sinensis* is an expensive fungus, owing to its low availability and the fact that it is difficult to culture. Therefore, *O. lanpingenesis* is of great importance to the management of traditional medicinal and natural supplemental needs. In the future, a more extensive metabolomics study of *O*. *lanpingensis* is recommended so its natural metabolites and their targeted pathways, which may be useful for the management of different diseases, can be more comprehensively identified. 

## Figures and Tables

**Table 1:** General features in the mitogenome of *O. lanpingensis*.

Genome Characteristics	*O. lanpingensis*
Genome size (base pairs)	117,565
G + C contents (%)	31.1
Protein coding genes (PCGs)	24
G + C contents of PCGs (%)	23.9
Structural proteins coding exons (%)	213
No rRNA/tRNAs (%)	2/23
G + C content of RNA genes (%)	35.66
rRNA + tRNA (%)	21.45
Coding regions (%)	82/47
Introns	35

**Table 2:** Summary of the gene content of the *O. lanpingensis* mitogenome.

Names of Genes	Number of Identified Genes in *O. lanpingensis*
Complex I/NADH dehydrogenase	Four genes: nad1, nad4, nad5, and nad6
Complex III/ubiquinol cytochrome c reductase	One gene: cob
Complex IV/cytochrome oxidase	Three genes: cox1, cox2, and cox3
ATP synthase	Three genes: atp6, atp8, and atp9
Ribosomal RNA (rRNA)	Two genes: rns and rnl
Transfer RNA (tRNA)	Twenty-five genes: trnT(aca), trnE(gaa), trnM(atg), trnM(atg), trnL2(tta), trnF(ttc), trnK(aaa), trnA(gca), trnL1(cta), trnQ(caa), trnH(cac), trnM(atg), trnR(cgt), trnC(tgc), trnR(aga), trnY(tac), trnD(gac), trnS1(agc), trnN(aac), trnG(gga), trnV(gta), trnI(atc), trnS2(tca), trnW(tga), and trnP(cca).

**Table 3:** Summary of genetic components of *O. lanpingensis* mitogenome.

Gene	Start	Stop	Strand	Length (AA)
rrnL	4464	5870	+	1407
rrnL	7501	8872	+	1372
trnT(aca)	9124	9195	+	72
trnE(gaa)	9873	9945	+	73
trnM(atg)	9949	10020	+	72
trnM(atg)	10023	10095	+	73
trnL2(tta)	10096	10178	+	83
trnF(ttc)	13739	13811	+	73
trnK(aaa)	14232	14304	+	73
trnA(gca)	14799	14871	+	73
trnL1(cta)	15315	15398	+	84
trnQ(caa)	15666	15739	+	74
trnH(cac)	15752	15826	+	75
trnM(atg)	16265	16336	+	72
nad2-0	26126	26551	+	426
nad3	26921	27259	+	339
atp9	27494	27676	+	183
cox2-0	30643	31092	+	450
cox2-1	33143	33214	+	72
trnR(cgt)	33266	33336	+	71
nad4l	33443	33655	+	213
nad5-0_a	36823	37263	+	441
nad6-1_b	37459	37746	−	288
nad6-1_a	37740	37871	−	132
nad5-0_b	38378	38767	+	390
nad5-1	41566	41889	−	324
cob-0_a	43042	43374	+	333
cob-1	46828	47022	+	195
cob-0_b	54216	54545	+	330
cob-0_c	56451	56741	+	291
trnC(tgc)	57176	57245	+	70
cox1-0_a	62859	63032	+	174
cox1-0_b	64480	64848	+	369
cox1-1	70853	71023	+	171
cox1-0_c	72504	72662	+	159
cox1-0_d	81521	81787	+	267
trnR(aga)	81988	82058	+	71
atp8-1	87591	87728	+	138
nad1_a	87995	88450	+	456
nad1_b	89767	90180	+	414
nad4	90966	92060	+	1095
atp8-0	93973	94182	+	210
atp6-1	94244	94585	+	342
atp6-0	95902	96354	+	453
rrnS	98327	99196	+	870
trnY(tac)	99649	99732	+	84
trnD(gac)	99755	99827	+	73
trnS1(agc)	100202	100282	+	81
trnN(aac)	100807	100878	+	72
cox3-1	104995	105120	+	126
cox3-0_a	106697	106903	+	207
cox3-0_b	108531	108788	+	258
trnG(gga)	110429	110499	+	71
nad6-0_a	110651	110818	+	168
nad6-0_b	112849	113208	+	360
trnV(gta)	115891	115963	+	73
trnI(atc)	116459	116530	+	72
trnS2(tca)	116828	116913	+	86
trnW(tga)	117024	117095	+	72
trnP(cca)	117410	117482	+	73

**Table 4:** Codon usage of PCGs in the mitogenome of *O. lanpingensis*.

Amino Acid	Codon	Number	Frequency of CodonUsage/1000	Fraction (%)
Ala	GCG	200	5.1	0.15
Ala	GCA	385	9.82	0.28
Ala	GCU	581	14.83	0.43
Ala	GCC	189	4.82	0.14
Cys	UGU	538	13.73	0.61
Cys	UGC	347	8.86	0.39
Asp	GAU	717	18.3	0.71
Asp	GAC	286	7.3	0.29
Glu	GAG	410	10.46	0.32
Glu	GAA	889	22.69	0.68
Phe	UUU	1689	43.1	0.76
Phe	UUC	538	13.73	0.24
Gly	GGG	294	7.5	0.22
Gly	GGA	409	10.44	0.3
Gly	GGU	487	12.43	0.36
Gly	GGC	171	4.36	0.13
His	CAU	474	12.1	0.66
His	CAC	248	6.33	0.34
Ile	AUA	1675	42.74	0.46
Ile	AUU	1444	36.85	0.39
Ile	AUC	543	13.86	0.15
Lys	AAG	890	22.71	0.28
Lys	AAA	2334	59.56	0.72
Leu	UUG	593	15.13	0.14
Leu	UUA	1635	41.72	0.39
Leu	CUG	313	7.99	0.07
Leu	CUA	738	18.83	0.18
Leu	CUU	699	17.84	0.17
Leu	CUC	239	6.1	0.06
Met	AUG	676	17.25	1
Asn	AAU	1620	41.34	0.72
Asn	AAC	633	16.15	0.28
Pro	CCG	120	3.06	0.12
Pro	CCA	283	7.22	0.27
Pro	CCU	430	10.97	0.42
Pro	CCC	199	5.08	0.19
Gln	CAG	312	7.96	0.32
Gln	CAA	666	17	0.68
Arg	AGG	518	13.22	0.23
Arg	AGA	957	24.42	0.43
Arg	CGG	147	3.75	0.07
Arg	CGA	204	5.21	0.09
Arg	CGU	216	5.51	0.1
Arg	CGC	173	4.41	0.08
Ser	AGU	717	18.3	0.24
Ser	AGC	508	12.96	0.17
Ser	UCG	197	5.03	0.07
Ser	UCA	570	14.55	0.19
Ser	UCU	661	16.87	0.23
Ser	UCC	274	6.99	0.09
Thr	ACG	200	5.1	0.11
Thr	ACA	619	15.8	0.35
Thr	ACU	650	16.59	0.37
Thr	ACC	303	7.73	0.17
Val	GUG	280	7.15	0.16
Val	GUA	752	19.19	0.42
Val	GUU	561	14.32	0.31
Val	GUC	200	5.1	0.11
Trp	UGG	353	9.01	0.35
Trp	UGA	661	16.87	0.65
Tyr	UAU	1699	43.36	0.74
Tyr	UAC	609	15.54	0.26
End	UAG	759	19.37	0.34
End	UAA	1504	38.38	0.66

**Table 5:** tRNAs in the mitogenome of *O. lanpingensis.*

AA	Anticodon	Number
Thr	TGT	One
Glu	TTC	One
Met	CAT	Three
Phe	GAA	One
Lys	TTT	One
Ala	TGC	One
Leu	TAG	One
His	GTG	One
Arg	ACG	One
Arg	TCT	One
Asp	GTC	One
Asn	GTT	One
Gly	TCC	One
Val	TAC	One
Ile	GAT	One
Ser	TGA	One
Sec	TCA	One
Pro	TGG	One

## Data Availability

Supplementary data can be provided on demand by the corresponding author.
